# Modeling craniofacial spliceosomopathies: a pathway toward deciphering disease mechanisms

**DOI:** 10.3389/fcell.2025.1624043

**Published:** 2025-09-26

**Authors:** Casey Griffin

**Affiliations:** Department of Molecular Pathobiology, College of Dentistry, New York University, New York, United States

**Keywords:** craniofacial spliceosomopathies, neural crest, spliceosome, *in vitro*, *in vivo*

## Abstract

Craniofacial spliceosomopathies are syndromes resulting from mutations in components of the spliceosome, presenting with facial dysostosis in combination with other phenotypes. An outstanding question in the field is how mutations in the ubiquitously expressed spliceosome lead to such cell- and tissue-specific disorders. To understand the etiology of these diseases and decipher the underlying mechanisms, scientists have turned to modeling these disorders in the laboratory. *In vivo* modeling of these disorders includes the use of mice, zebrafish, and frogs, whereas *in vitro* modeling typically uses embryonic stem cells (ESCs) and induced pluripotent stem cells (iPSCs). The goal with these models is to recapitulate the human disorders in a manner that is conducive to scientific exploration. In this review, we briefly describe the major craniofacial spliceosomopathies and discuss recent advances using model systems that have helped understand the root cause of these conditions.

## Introduction

The spliceosome is a complex of RNA and proteins that functions to process pre-messenger RNA (pre-mRNA) into mRNA by identifying introns, splicing them out, and joining the exons. The steps of pre-mRNA splicing are as follows: (1) 5′ intron recognition, (2) 3′ intron recognition, (3) pre-catalytic spliceosome recruitment, (4) catalytic activation, and (5) exon joining (Will and Lührmann, 2011; Griffin and Saint-Jeannet, 2020). The major spliceosome is made up of five U subunits: U1, U2, U4, U5, and U6, which are each composed of small nuclear RNAs (snRNAs) associated with small nuclear ribonucleoproteins (snRNPs) and other proteins (Will and Lührmann, 2011). In the minor spliceosome, which is involved in the recognition of rare introns (Verma et al., 2018), the U2 subunit is replaced by the U12 snRNA.

Mutations in any of the components of the spliceosome can give rise to diseases known as spliceosomopathies. Although the spliceosome is active in all cells of the body to process pre-mRNA, most spliceosomopathies are cell- or tissue-specific in their manifestation and, as such, represent a conundrum in the field to understand the mechanism underlying these pathologies. The four major classes of spliceosomopathies are retinitis pigmentosa, myelodysplastic syndromes, cancers, and craniofacial spliceosomopathies (Griffin and Saint-Jeannet, 2020). Retinitis pigmentosa is a genetic disorder characterized by the deterioration of the photoreceptors of the retina, which can result in blindness (Hamel, 2006). Myelodysplastic syndromes are disorders in which there is defective hematopoiesis, affecting one or more hematopoietic lineages (Catenacci and Schiller, 2005). Many cancers can be caused by mutations in spliceosome components; aberrant splicing events have been linked to cancer proliferation, invasion, and metastasis (Mrid et al., 2025; Cao and Li, 2024; Bak-Gordon and Manley, 2025; Hermán-Sánchez et al., 2024; Stanley and Abdel-Wahab, 2022, and many more). Craniofacial spliceosomopathies are disorders in which mutations of the spliceosome cause defects in the skeletal elements of the craniofacial complex, more specifically, the neural crest-derived skeletal elements of the face (Lehalle et al., 2015).

The neural crest is an embryonic cell population that derives from the neural plate border as epithelial cells, undergoes an epithelial-to-mesenchymal transition, and then migrates through the pharyngeal arches to give rise to a variety of cell types, including the craniofacial skeleton. Although craniofacial spliceosomopathies cover a wide range of phenotypes and manifestations, they all share defects in the neural crest-derived structures of the face. In this review, we focus on craniofacial spliceosomopathies and the models that have been developed to study them, with the goal of discovering why mutations in the ubiquitously active spliceosomal complex give rise to such phenotypically specific disorders.

## Craniofacial spliceosomopathies

**FIGURE 1:**
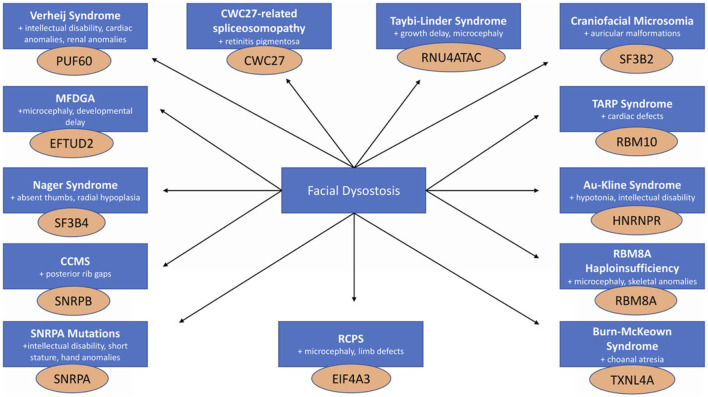
Craniofacial spliceosomopathies are all characterized by facial dysostoses that present in combination with a diverse array of phenotypes arising from mutations in genes that encode proteins of the spliceosome.

Although craniofacial spliceosomopathies are rare diseases, they belong to the category of facial dysostoses, which represent one-third of all live births with congenital anomalies (Trainor and Andrews, 2013). The craniofacial component of these diseases often occurs in combination with other phenotypes (Figure 1; Table 1). Many of these disorders are due to loss-of-function mutations in genes that encode for spliceosomal proteins, making many of the patients haploinsufficient. The common features of these disorders are malformations of the derivates of the first and second pharyngeal arches, which occur during embryogenesis (Trainor and Andrews, 2013). These defects are typically considered maxillary, malar, and mandibular hypoplasia, cleft palate, and outer and/or middle ear defects. In particular, the skeletal defects seen in these disorders are developmental in nature and are mostly due to impairment of the neural crest. The majority of these disorders are non-lethal, with the current treatment involving reconstructive surgeries to ease pain and improve cosmetics, usually beginning at birth and continuing throughout adolescence and adulthood. Diagnostic criteria for these disorders include clinical assessment and genetic testing.

**TABLE 1:** List of genes causing craniofacial spliceosomopathies, characteristics of these genes, and types of models available.

Gene	Role in spliceosome	Mode of inheritance	Pathophysiological mechanism	Pathophysiology	Model
PUF60	3′ splice-site recognition	Mostly *de novo* and rarely autosomal dominant	Haploinsufficiency	Neurodevelopmental delay, intellectual disability, brain malformations, microcephaly, short stature, and ocular, craniofacial, skeletal, cardiac, and renal anomalies	None
EFTUD2	U5 snRNP	75% *de novo* and 25% dominant inheritance	Haploinsufficiency	Craniofacial malformations, microcephaly, developmental delay, dysmorphic appearance, choanal atresia, sensorineural hearing loss, and cleft palate	Mouse, fish, frog, and human cells
SF3B4	U2 and U12 snRNPs	*De novo* and autosomal dominant	Haploinsufficiency	Midface retrusion, micrognathia, absence of thumbs, and radial hypoplasia	Mouse, fish, frog, and human cells
SNRPB	Sm ring in the U1, U2, U4, and U5 subunits	*De novo* and autosomal dominant	Haploinsufficiency	Micrognathia, glossoptosis, cleft palate, and posterior rib gaps	Mouse and frog
SNRPA	U1 snRNP	*De novo*	Haploinsufficiency	Intellectual disability, short stature, and minor craniofacial and hand anomalies	None
EIF4A3	Exon junction complex	Autosomal recessive	Repeat expansion	Craniofacial malformations with microcephaly and limb defects	Mouse and frog
TXNL4A	U5 snRNP	Autosomal recessive	Haploinsufficiency	Choanal atresia, hearing loss, cleft lip/palate, and other craniofacial anomalies	Frog and human Cells
RBM8A	Exon junction complex	Autosomal recessive	Haploinsufficiency	Microcephaly, facial gestalt, cleft lip/palate, and skeletal anomalies	None
HNRNPK	C complex	*De novo*	Haploinsufficiency	Hypotonia, intellectual disability, and typical facial features	None
RBM10	A complex	*De novo* or X-linked dominant	Haploinsufficiency	Cleft palate, Talipes equinovarus, atrial septal defect, Robin sequence, and persistent left superior vena cava	Human cells
SF3B2	U2 snRNP	*De novo* and autosomal dominant	Haploinsufficiency	Auricular malformations, underdevelopment of the mandible, and effects on middle ear ossicles, temporal bone, zygoma, and cranial nerves	Frog
RNU4ATAC	snRNA involved in minor intron splicing	Autosomal recessive	Haploinsufficiency	Growth delay, microcephaly, intellectual deficiency, and bone abnormalities	Fish and human cells
CWC27	Spliceosome-associated cyclophilin	Autosomal recessive	Haploinsufficiency	Retinitis pigmentosa and craniofacial abnormalities	Mouse

### Verheij syndrome

Verheij syndrome (OMIM #615583) involves a spectrum of phenotypes, including neurodevelopmental delay, intellectual disability, brain malformations, microcephaly, short stature, and ocular, craniofacial, skeletal, cardiac, and renal anomalies (Fennell et al., 2022). This syndrome is caused by a deletion in the 8q24.3 region, where the *PUF60* gene is located (Verheij et al., 2009; Miao et al., 2024). *PUF60* is involved in 3′ splice-site recognition, interacting with U2AF in RNA binding and splicing activation (Hoogenboom et al., 2024; Hastings et al., 2007). The majority of cases of Verheij syndrome are due to *de novo* mutations; however, rare cases show an autosomal dominant inheritance pattern (Verheij et al., 2009; Sivasubramanian and Ayyavoo, 2024). Haploinsufficiency of *PUF60*, due to deletions spanning from 78 kb to 1 Mb, has been found to be the driver of Verheij syndrome, with the copy number variants (CNVs) affecting the dose of multiple genes depending on the size of the deletion (Hoogenboom et al., 2024; Dauber et al., 2013).

### Mandibulofacial dysostosis, Guion-Almeida type

Mutations in *EFTUD2*, part of the U5 snRNP of the spliceosome, have been identified as the cause of mandibulofacial dysostosis, Guion-Almeida type (MFDGA; OMIM #610536) (Lines et al., 2012; Beauchamp and Jerome-Majewska, 2024). MFDGA is characterized by craniofacial malformations, microcephaly, developmental delay, and dysmorphic appearance but may also include choanal atresia, sensorineural hearing loss, and cleft palate (Guion-Almeida et al., 2006; Wieczorek et al., 2009). The frequency of MFDGA is unknown, with approximately 100 affected individuals identified so far, harboring 86 distinct *EFTUD2* mutations (Beauchamp et al., 2020). Most mutations are stop-gain and splicing mutations, with roughly 75% of patients harboring *de novo* mutations, whereas dominant inheritance is observed in the remaining patients (Huang et al., 2016).

### Nager and Rodriguez syndromes

Nager syndrome (OMIM #154400) is a type of acrofacial dysostosis characterized by midface retrusion, micrognathia, absence of thumbs, and radial hypoplasia (Bernier et al., 2012; Czeschik et al., 2013; Petit et al., 2014). Sixty percent of patients with Nager syndrome have mutations in *SF3B4*, with haploinsufficiency of *SF3B4* being the underlying cause of the disorder (Bernier et al., 2012). Rodriguez syndrome (OMIM #201170) is also caused by mutations in *SF3B4* (Drivas et al., 2019). The patients have similar features as Nager syndrome patients; however, the phenotype is typically more severe and involves lower limb and cardiac defects (Rodríguez et al., 1990). *SF3B4* encodes for SAP49 and is part of the U2 and U12 snRNPs (Will and Lührmann, 2011), functioning in 3′ branchpoint sequence recognition. The frequency of Nager syndrome is unknown, with approximately 100 cases found worldwide.

### Cerebro-costo-mandibular syndrome

Mutations in *SNRPB* cause cerebro-costo-mandibular syndrome (CCMS; OMIM #117650), which is a disorder that includes micrognathia, glossoptosis, cleft palate, and posterior rib gaps (Lynch et al., 2014). *SNRPB* is part of the Sm ring that is the scaffold for snRNPs in the U1, U2, U4, and U5 subunits (Schwer et al., 2016; Zahoor et al., 2024). Mutations tend to be heterozygous regulatory mutations, with high frequency of mutation in the premature termination codon-containing exons. CCMS is a rare disease, with approximately 80 reported cases (Bacrot et al., 2015).

### Mutations in SNRPA

Mutations in gene *SNRPA* (OMIM #182285) lead to a yet unnamed syndrome that includes intellectual disability, short stature, and minor craniofacial and hand anomalies (Rangel-Sosa et al., 2018). The mutations are homozygous missense variants in *SNRPA*, which encodes for an snRNP in the U1 subunit of the spliceosome (Nelissen et al., 1991). Mutations tend to be localized to the first 10–89 amino acids, which is the domain associated with RNA binding (Rangel-Sosa et al., 2018; Jessen et al., 1991).

### Richieri-Costa–Pereira syndrome

Richieri-Costa–Pereira syndrome (RCPS; OMIM #268305) is a type of acrofacial dysostosis, in which patients exhibit craniofacial malformations with microcephaly and limb defects (Bertola et al., 2018; Favaro et al., 2014; Hsia et al., 2018). This disorder is due to decreased expression levels of *EIF4A3*, a member of the exon junction complex in the spliceosome (Le Hir et al., 2016). The decreased levels of this gene are attributed to increased repeats in the 5′UTR of the gene. RCPS is a rare disorder, with less than 50 published cases (Pardo et al., 2021), with a possible phenotypic spectrum related to the number of repeats in the gene (Bertola et al., 2018).

### Burn–McKeown syndrome

Mutations in *TXNL4A* cause a disorder known as Burn–McKeown syndrome (BMKS; OMIM #608572). This condition is characterized by choanal atresia, hearing loss, cleft lip/palate, and other craniofacial anomalies (Wieczorek et al., 2014). *TXNL4A* encodes a component of the spliceosome U5 snRNP, and the mutations in patients lead to reduced expression and ultimately reduced assembly of the snRNP complex. This disease has been found in 20 individuals with biallelic pathogenic variants (Lüdecke and Wieczorek, 2022). Most patients have a loss-of-function deletion in the promoter region of the gene; however, patients have been identified with intronic deletions (Wood et al., 2022).

### RBM8A haploinsufficiency/1q21.1 deletion syndrome

Mutations in *RBM8A*, a member of the exon junction complex, resulting in haploinsufficiency lead to a disorder characterized by microcephaly, facial gestalt, cleft lip/palate, and skeletal anomalies (OMIM #274000) (Gamba et al., 2016; Mao et al., 2015). These mutations also involve microdeletions of the 1q21.1 chromosome, resulting in variable syndromic phenotypes (Upadhyai et al., 2020). Occurrence of CNVs in 1q21.1 is rare, with less than 40 reports in the literature (Brunetti-Pierri, et al., 2008).

### Au–Kline syndrome

Au–Kline syndrome (OMIM #616580) is a developmental disorder characterized by hypotonia, intellectual disability, and typical facial features (Au et al., 2019). This disorder is due to variants in *HNRNPK*, which is part of the spliceosome C complex, resulting in impairment of *Hox* gene expression (Duijkers et al., 2019). Loss-of-function mutations are also associated with a specific DNA methylation signature (Choufani et al., 2022). All currently known patients have *de novo* mutations, with some including missense variants and others being deletions of 9q21.32, encompassing *HNRNPK* (Au et al., 2018).

### TARP syndrome

Mutations in *RBM10* cause an X-linked form of cleft palate known as TARP syndrome (Talipes equinovarus, Atrial septal defect, Robin sequence, and Persistent left superior vena cava; OMIM #311900) (Johnston et al., 2010; Gripp et al., 2011). *RBM10* is an RNA-binding protein that plays a role in the A complex of the spliceosome, regulating alternative splicing. TARP syndrome is a very rare disorder, with approximately 30 cases reported (Omorodion et al., 2023). Mutations tend to be loss-of-function, occurring either *de novo* or via X-linked dominant inheritance, in which male children are affected and mothers may present some mosaicism (Johnston et al., 2013).

### Craniofacial microsomia/oculo-auriculo-vertebral spectrum/ Goldenhar syndrome

Craniofacial microsomia (OMIM #164210) is a disorder that includes auricular malformations and underdevelopment of the mandible but may also affect the middle ear ossicles, temporal bone, zygoma, and cranial nerves (Beleza-Meireles et al., 2014; Keogh et al., 2007; Timberlake et al., 2021). The most prevalent genetic cause of craniofacial microsomia is haploinsufficiency of SF3B2, a component of the U2 small nuclear ribonucleoprotein complex. Loss-of-function mutations in SF3B2 account for 3% of sporadic cases and 25% of familial cases, with mutations spread across the entirety of the gene (Timberlake et al., 2021). Craniofacial microsomia occurs in between 1 in 5,600 and 1 in 26,550 births, but mild cases are often difficult to diagnose (Gougoutas et al., 2007).

### Taybi–Linder, Roifman, and Lowry–Wood syndromes

Taybi–Linder syndrome (TALS; OMIM #210710), or microcephalic osteodysplastic primordial dwarfism type I (MOPD1), is characterized by severe growth delay, microcephaly, intellectual deficiency, bone abnormalities, and other factors ultimately resulting in early mortality (Hagiwara et al., 2021). Roifman syndrome (OMIM #616651) is a disorder characterized by growth retardation, cognitive delay, and spondyloepiphyseal dysplasia (Merico et al., 2015). Lowry–Wood syndrome (OMIM #226960) is a similar disorder characterized by multiple epiphyseal dysplasia, microcephaly, and intellectual disability (Farach et al., 2018). All three of these disorders are attributed to mutations in RNU4ATAC, a small nuclear RNA essential for minor intron splicing (Edery et al., 2011).

### CWC27-related spliceosomopathy

The *CWC27*-related spliceosomopathy (OMIM #250410) is also known as retinitis pigmentosa with or without skeletal anomalies. Although this is mainly categorized as a retina disorder, when the patients have skeletal anomalies, they include craniofacial abnormalities, classifying this disorder also as a craniofacial spliceosomopathy (Xu et al., 2017). Variants of *CWC27* are diverse and may result from missense mutations, nonsense mutations, splice-site variants, small insertions, small deletions, and gross deletions (Li et al., 2024).

Craniofacial spliceosomopathies represent a broad array of phenotypes affecting many parts of the body; however, they all have one common denominator: defects in the neural crest-derived craniofacial skeleton, and can be categorized as facial dysostoses (Figure 1). Although the splicing factors affected under these conditions are found across the spliceosome and carry distinct functions (Griffin and Saint-Jeannet, 2020), they all cause a similar craniofacial phenotype, pointing at a possible common root cause and driving the need to further investigate the underlying mechanisms of these disorders.

## Modeling craniofacial spliceosomopathies

**FIGURE 2:**
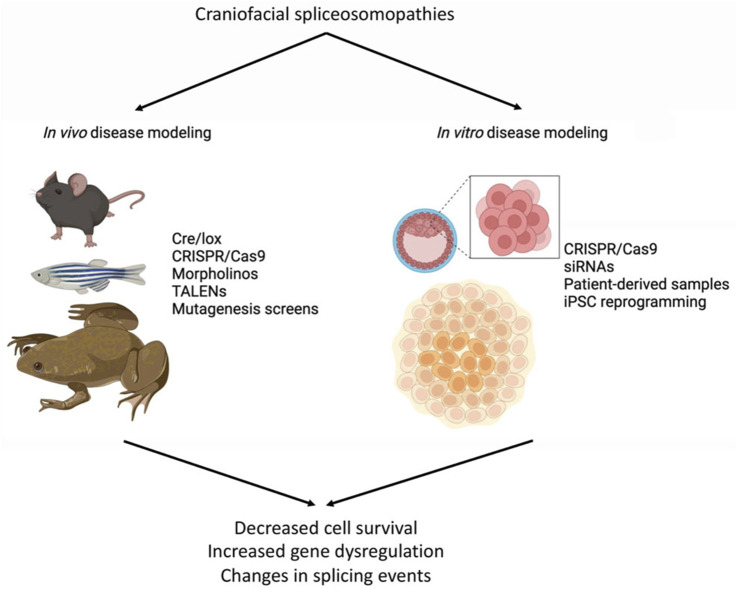
Tools available for *in vivo* and *in vitro* disease modeling.

The etiology of congenital diseases can be studied through *in vivo* or *in vitro* modeling, which are expected to closely duplicate these human conditions (Figure 2). Preferred *in vivo* models include mouse (*Mus musculus*), zebrafish (*Danio rerio*), and frog (*Xenopus laevis* or *Xenopus tropicalis*), with tools such as CRISPR/Cas9, Cre/lox, TALENs, and mutagenesis screens to target specific genes and/or mutations. Morpholino antisense oligonucleotides are also used in fish and frogs for gene knockdowns (Figure 2). For *in vitro* modeling, mouse or human embryonic stem cells (ESCs) or induced pluripotent stem cells (iPSCs) from patient samples are commonly used. Gene function can be manipulated by small interfering RNA (siRNA), short hairpin RNA (shRNA), or CRISPR/Cas9 to engineer disease-causing mutations, allowing for the investigation of the consequences of these mutations on cellular processes such as proliferation, apoptosis, migration, and differentiation (Figure 2). In this section, current models available for studying craniofacial spliceosomopathies are summarized (Table 2), with an emphasis on how these tools can be used to understand the underlying mechanisms of these diseases.

**TABLE 2:** List of craniofacial spliceosomopathies and the models available to study them.

Disease	Gene affected		Disease model	
		Mouse	Fish	Frog
Mandibulofacial dysostosis, Guion-Almeida type	EFTUD2	Beauchamp et al. (2019); Beauchamp et al. (2021); Beauchamp et al. (2022)	Deml et al. (2015); Lei et al. (2016)	Park et al. (2022)
Nager syndrome	SF3B4	Yamada et al. (2020); Kumar et al. (2023); Kumar et al. (2024)	Ulhaq et al. (2023); Ulhaq et al. (2024)	Devotta et al. (2016); Griffin et al. (2025)
Cerebro-costo-mandibular syndrome	SNRPB	Alam et al. (2022)		Park et al. (2022)
Acrofacial dysostosis Richieri-Costa–Pereira syndrome	EIF4A3	Lupan et al. (2023)		Haremaki et al. (2010)
Burn–McKeown syndrome	TXNL4A			Park et al. (2022)
TARP syndrome	RBM10			
Craniofacial microsomia/OAVS/Goldenhar syndrome	SF3B2			Timberlake et al. (2021)
Taybi–Linder, Roifman, and Lowry–Wood Syndromes	RNU4ATAC		Khatri et al. (2023)	

### Mouse models

Mouse models are a hallmark of disease modeling and can be a powerful system for understanding disease mechanisms and phenotypes, when the model actually represents the disease in a clinically relevant manner.

Conditional knockout of *Snrpb* in the brain and neural crest lineages using the *Wnt1-Cre2* driver was used to model CCMS. The heterozygous mutant embryos (Snrpb^ncc+/−^) recapitulate the disease, showing craniofacial hypoplasia with decreased differentiation of craniofacial cartilage and bone, and reduced postnatal survival (Alam et al., 2022). Although most neural crest cells form in the head and migrate into the pharyngeal arches in the mutants, a subset of neural crest cells undergo apoptosis, indicating that increased neural crest cell death accounts for aspects of this disease. Snrpb^ncc+/−^ embryos at E9.0 also had many significantly altered splicing events compared to the wild type, with the most abundant being skipped exons and retained introns. Among these, 13 transcripts required for craniofacial development, including *Rere*, *Dyrk2*, and *Pou2f1*, were identified as having increased exon skipping, potentially contributing to the craniofacial defects observed in the mutant embryos (Alam et al., 2022).

The *Sf3b4* ^*ncc/ncc*^ and *Sf3b4* ^*ncc/−*^ mice with loss of *Sf3b4* in neural crest cells is another useful model for Nager and Rodriguez syndromes (Kumar et al., 2024). This conditional knockout was able to recapitulate the craniofacial and cardiac phenotype observed in patients. Similarly, *Eftud2* ^*ncc−/−*^ mouse was generated to recapitulate MFDGA *in vivo*. Although these mice exhibited craniofacial malformations, they did not survive until birth; however, this model was still used to understand the connection between *Eftud2* and the P53 pathway (Beauchamp et al., 2021; Beauchamp et al., 2022). In particular, exon skipping and increased levels of an alternatively spliced form of Mdm2, a p53 pathway gene, were found in mouse embryos. Treatment of mutant embryos with an inhibitor of p53 (pifithrin-a) ameliorated the craniofacial abnormalities found in the untreated embryos, connecting increased p53 activity to the mechanism of MFDGA.

The mouse, however, is not always the best system for modeling craniofacial spliceosomopathies. Frequently, homozygous knockouts of splicing factors are embryonic lethal, whereas heterozygous knockouts show no craniofacial phenotype. Such is the case for the *Eftud2* CRISPR/Cas9 knockout mouse, in which there is no survival post-implantation, with the heterozygotes failing to model MFDGA (Beauchamp et al., 2019). Similarly, the *Sf3b4* heterozygous knockout mouse does not show any craniofacial phenotype and instead shows defects in the axial skeleton and the forebrain (Yamada et al., 2020), accompanied by mis-splicing of chromatin remodelers and dysregulation of *Hox* gene expression (Kumar et al., 2023). In the case of *CWC27*, mutant mice show the retinal degeneration phenotype of the associated disorder, but no craniofacial malformations are described in either of two mutant models—*Cwc27* ^*K338fs/K338fs*^ and *Cwc27Tm1a/K338fs* (gene trapping of exon 3 and CRISPR/Cas9-mediated frameshift compound heterozygote) (Bertrand et al., 2022; Lu et al., 2023).

The best approach to study spliceosomopathies in mice appears to be the use of conditional knockouts, as demonstrated with *Eftud2* and *Sf3b4* using the *Wnt1-Cre2* driver. However, this limits the tissues in which the defects can be examined when multiple tissues are affected. For example, conditional knockout of *Eif4a3* in the radial glial cells allows for the examination of the microcephaly phenotype of patients but disregards any analysis of the craniofacial malformations observed in RCPS (Lupan et al., 2023). Therefore, considering other models in addition to the mouse may be beneficial.

### Zebrafish models

Zebrafish is a popular vertebrate model system, recognized for the ease at making transgenic animals and imaging analysis due to the transparency of the embryos. Mutant embryos can be generated in a number of ways in zebrafish, with tools lending themselves to the specific attributes of a gene or disease. For example, morpholino antisense oligonucleotides can be used to target specific genes and knockdown their function. Such is the case with *RNU4ATAC*, in which morpholino-mediated knockdown resulted in defects in primary cilia, such as decreased number and function, thereby recapitulating the phenotype of TALS-patient fibroblasts (Khatri et al., 2023). TALEN-mediated disruption has also been used to induce mutations in zebrafish. This was done for a truncation mutation in the *eftud2* gene to mimic a mutation found in a MFDGA patient. Mutants displayed a small head and small eye, identifying novel eye phenotypes possibly associated with MFDGA (Deml et al., 2015). A separate mutant construct of *eftud2* known as the *fn10a* mutant has been generated from a mutagenesis screen, and this mutant is useful for studying the impact on neurogenesis (Lei et al., 2016). In this model, neural progenitors experience increased apoptosis and mitosis, coupled with splicing deficiencies including increased retained intron and exon skipping in genes enriched for several KEGG pathways such as “cell cycle,” “p53 signaling pathway,” and “spliceosome.” However, this mutant lacks the craniofacial phenotype of MFDGA.

Unfortunately, similar to the mouse, zebrafish models might lack certain characteristics of craniofacial spliceosomopathies observed in patients. For example, sf3b4^−/−^ mutant zebrafish show no craniofacial malformations but instead exhibit features of retinitis pigmentosa (Ulhaq et al., 2023; Ulhaq et al., 2024). Although retinitis pigmentosa is a spliceosomopathy (Griffin and Saint-Jeannet, 2020), it has not clinically been attributed to mutations in *Sf3b4*, and Nager syndrome patients do not show clinical signs of retinitis pigmentosa. Further studies will define the clinical relevance of this model.

### Frog models

*Xenopus* is an excellent model system for studying developmental disorders because of the ease at which key developmental processes can be observed and manipulated. A tool broadly used in *X. laevis* is morpholino antisense oligonucleotides to knockdown gene function during development. For example, a comparative study has been performed by knocking down individual splicing factors—namely, *eftud2*, *snrpb*, and *txnl4a*, which have been linked to MFDGA, CCMS, and BMKS, respectively—and analyzing the consequences on neural crest and craniofacial development. The main results indicate that neural crest progenitor formation is similarly affected in each knockdown through a mechanism that involves increased apoptosis and results in hypoplastic craniofacial cartilages (Park et al., 2022). A study using a morpholino against *eif4a3* found that loss of this protein function disrupts derivatives of the neural plate and neural plate border, including some neural crest derivatives (although the craniofacial structure is unaffected) (Haremaki et al., 2010). Similar morpholino studies to interfere with *sf3b4* or *sf3b2* function have shown shared mechanisms underlying these phenotypes, characterized by impaired neural crest formation, coupled with an increase in apoptosis in the head region and reduced craniofacial cartilages, perhaps hinting at a common root cause to some, if not all craniofacial spliceosomopathies (Devotta et al., 2016; Timberlake et al., 2021).

The *X. laevis* allotetraploid genome and the relatively long generation time (10–12 months) render genetic analysis challenging in this organism. In recent years, the related species *X. tropicalis* has become more broadly used. It offers the same embryological advantages as its allotetraploid counterpart, with a shorter generation time (5–7 months) and a diploid genome. *Xenopus tropicalis* has been used to generate CRISPR/Cas9 knockout mutant lines, which enable a more consistent knockout than morpholinos, reducing off-target effects and allowing for large-scale genomic analysis. Recently, an *Sf3b4* knockout mutant line was generated, and it was found that the homozygous null embryos showed reduced neural crest cell migration, increased apoptosis in the head region, and decreased craniofacial cartilage precursors (the heterozygous embryos were comparable to the wild type). These phenotypes were reflected in dysregulated genes as revealed by bulk RNA-sequencing, with downregulated genes categorized into GO terms such as “neural crest cell migration,” “extracellular matrix organization,” and “negative regulation of extrinsic apoptotic signaling.” These dysregulated genes were preceded in developmental time by mis-splicing events, predominately increased abnormal skipped exons, for genes categorized into GO terms such as “RNA splicing,” “regulation of embryonic development,” and “regulation of apoptotic process” (Griffin et al., 2025). Further studies will use this information to identify the gene networks and pathways affected under this craniofacial condition. These studies show that frogs provide a unique system for studying craniofacial spliceosomopathies within the context of development.

Animal models are extremely powerful tools to investigate disease mechanisms. However, there are also downsides to working with these models. Most animal models require a complete gene dosage reduction to recapitulate the disease, taking away from the ability to exactly replicate the human conditions, which are often haploinsufficient. Moreover, the reliance on conditional mutations to model a desired phenotype restricts the ability to interrogate the role of a gene in a broad range of cell and tissue types. Finally, animal models are inherently different than humans and, therefore, may introduce variables that are not directly relevant to the human diseases. Therefore, turning to *in vitro* modeling may help support and expand the findings of *in vivo* studies.

### *In vitro* modeling

*In vitro* modeling of craniofacial spliceosomopathies is an emerging field, with only a small number of disorders examined so far. The use of relevant cell lines, whether primary or engineered, especially those of human origin, holds great potential for elucidating the underlying mechanisms of these diseases in terms of gene function.

For example, the use of mouse mandibular MEPA (mouse embryonic pharyngeal arch) cell lines to examine the role of *RBM10* in TARP syndrome has allowed for the identification of *RBM10*-binding sites in the genome and the elucidation of its role in regulating alternative splicing (Rodor et al., 2016). This group also used the MEPA cells to generate an *RBM10* CRISPR/Cas9 knockout cell line to characterize the phenotype at the cellular level, which highlights that loss of *RBM10* leads to proliferation defects and changes in the differentiation potential of mutant cells. Human cell line HEK293 has been used to generate an *EFTUD2* CRISPR/Cas9 knockout cell line, with a heterozygous loss-of-function mutation that is a null allele equivalent to MFDGA patient mutations (Wood et al., 2019). This cell line was used to identify diminished proliferation, increased sensitivity to endoplasmic reticulum (ER) stress, and mis-expression of ER stress response genes as the potential underlying mechanisms of MFDGA. Another study used primary cells—fibroblasts from TALS patients—to understand the role of *RNU4ATAC* in the disorder and compare function to the *in vivo* phenotypes (Khatri et al., 2023). The work indicates alterations in primary cilium function in these cells, which reflected phenotypes observed *in vivo*, thereby demonstrating the strength of this *in vitro* model in recapitulating some aspects of the disease.

The use of human embryonic stem cells (hESCs) and iPSCs to model diseases has grown exponentially over the recent years. These cells offer the ability to use human samples to investigate the manifestation of disorders in specific cell types or in tissues in the form of organoids. Recently, hESCs have been used to investigate the underlying mechanism of Nager syndrome. Taking advantage of a well-described protocol to derive neural crest cells from hESCs (Bajpai et al., 2010), it is possible to specifically investigate the function of SF3B4 in differentiating neural crest cells. siRNA-mediated knockdown of *SF3B4* revealed a requirement for SF3B4 in neural crest cell production, survival, and differentiation (Griffin and Saint-Jeannet, 2025), showing some parallels with the corresponding animal models (Devotta et al., 2016; Kumar et al., 2024; Griffin et al., 2025). Similarly, BMKS patient iPSCs have been used to investigate the differentiation potential and behavior of neural crest cells with reduced *TXNL4A* expression (Wood et al., 2020). TXNL4-deficient cells exhibited defective differentiation into neural crest cells, with significant differences in neural border and neural crest marker genes, a delay in the epithelial-to-mesenchymal transition, and dampened response to WNT signaling, an important regulator of craniofacial development (Wood et al., 2020). RCPS patient-derived iPSCs have been used to generate cortical organoids to study neurogenesis with *EIF4A3* haploinsufficiency (Lupan et al., 2023). Coupled with *in vivo* mouse work, it was determined that *EIF4A3* mediates neurogenesis by controlling mitosis and cell survival; with reduction in *EIF4A3*, there is extensive cell death and impaired neurogenesis.

*In vitro* modeling of diseases allows for the use of human samples to interrogate the mechanisms of the disorders in the context of the patient mutations and/or specific cell types that are affected. In the case of craniofacial spliceosomopathies, hESCs and iPSCs can be differentiated into neural crest cells and their derivatives, allowing for examination of disease-causing mutations in the cells that are primarily affected in these patients. However, there are also some limitations to *in vitro* disease modeling, such as the limited number of cell types differentiating in a dish that does not fully capture the complexity of tissues *in vivo*; future technological advances such as 3D organoids may help alleviate some of these shortcomings. Another limitation is the difficulty of obtaining samples from patients with such rare diseases.

## Conclusions and perspectives

In this paper, we summarize and discuss the models developed to understand the etiology of several craniofacial spliceosomopathies. Although these models have started to narrow down some of the key mechanisms underlying these diseases, which include increased apoptosis and dysregulated gene expression and splicing events, important gaps remain to be addressed to better understand what makes neural crest cells a preferred target in these pathologies. It is also important to point out that several craniofacial spliceosomopathies have not yet benefited from *in vivo* or *in vitro* modeling, which includes Verheij syndrome (*PUF60*), mutations in *SNRPA*, *RBM8A* haploinsufficiency, and Au–Kline syndrome (*HNRNPK*). It is essential that this group of diseases be studied at a global level to obtain a comprehensive understanding of the underlying pathomechanisms.

It is intriguing that most models of craniofacial spliceosomopathies *in vivo* do not fully replicate the human diseases in their presentation. Furthermore, animal models typically require homozygosity to develop the phenotype, whereas the majority of craniofacial spliceosomopathies are found to be heterozygous mutations in patients. Perhaps, this is due to some compensatory underlying mechanisms in these organisms’ spliceosomes that have been lost or are lacking in humans. Either way, it makes studying these diseases more challenging because the systems must be manipulated in ways that may affect downstream mechanistic studies.

Important efforts are currently underway in the field to develop *in vitro* models using patient-derived cells or genome-edited cells that reflect patient mutations. These cells can be obtained from patients directly and studied as primary cells or be reprogrammed into iPSCs. Alternatively, hESCs or iPSCs can be edited with CRISPR/Cas9 to induce patient mutations. The derived stem cells can then be differentiated into neural crest cells using defined protocols, testing the impact of the mutations on neural crest generation, differentiation potential, and survival, with the added potential for transcriptomic and proteomic analyses. Eventually, neural crest cells will need to be incorporated into 3D organoids with other cell types to reproduce the *in vivo* patient environment more closely.

Overall, disease modeling is an important tool to understand the etiology of understudied disorders. Although all model systems have limitations, it is critical to use them in combination to develop the most comprehensive understanding of the mechanisms underlying these conditions.
